# Methane Yield and Microbial Dynamics in Solid-State Anaerobic Codigestion of Sugar Cane Bagasse and Bovine Manure

**DOI:** 10.1021/acsomega.5c08172

**Published:** 2026-02-19

**Authors:** Larissa Maria Silveira Pereira, Carlos Vitor Ribeiro Pereira, Ilton José Baraldi, Igor Vinicius Machado Sophiatti, Flaviane Eva Magrini, Suelen Paesi, Prasad Kaparaju, Thiago Edwiges

**Affiliations:** † 74354 Federal University of Technology—Paraná (UTFPR), Avenida Brasil 4232, Parque Independência, Medianeira 85884-000, Brazil; ‡ Molecular Diagnostic Laboratory, Biotechnology Institute, 58802 University of Caxias do Sul (UCS), Caxias do Sul 95070-560, Brazil; § 5723 Griffith University, 170 Kessels Rd, Nathan, Queensland 4111, Australia

## Abstract

Solid-state anaerobic digestion (SS-AD) offers a sustainable route for the valorization of agro-industrial residues, yet its efficiency is often constrained by limited mass transfer and process instability. This study investigated the codigestion of sugar cane bagasse (SCB) and bovine manure (BM) under varying substrate-to-inoculum (S/I) ratios (0.5–2.0), total solids (TS, 8.9–17.2%), and carbon-to-nitrogen (C/N, 16–37) ratios to identify optimal operating conditions for methane production. The highest volumetric methane yields (12.6 to 13.9 L CH_4_ L^–1^ reactor) occurred at intermediate TS (11.6 to 12.5%) and C/N (19 to 26%) values (T1 and T4). Monodigestion of SCB was feasible up to S/I = 1.0, while codigestion with BM enabled higher organic loading (S/I = 1.5) without inhibition. A shift in the microbiological profile was observed from T1 to T4, with a predominance of *Clostridium sensu stricto 1* and *Solibacillus*, both of which are associated with the degradation of lignocellulosic compounds. In T4, the presence of Pseudomonas stood out, indicating a greater microbial diversity in this SCB monodigestion trial. The archaeal community was dominated by *Methanosarcina* in T4, with a higher diversity of genes associated with the hydrogenotrophic pathway, followed by the acetoclastic pathway. By linking microbial community dynamics to process performance, this study defines operational thresholds that enhance SS-AD efficiency and resilience, supporting the development of robust dry-digestion systems for lignocellulosic biomass valorization.

## Graphical abstract

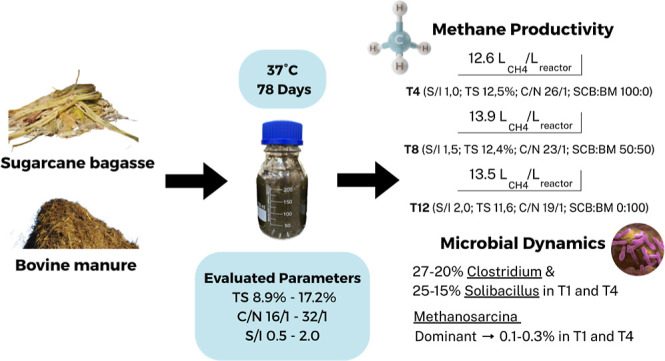

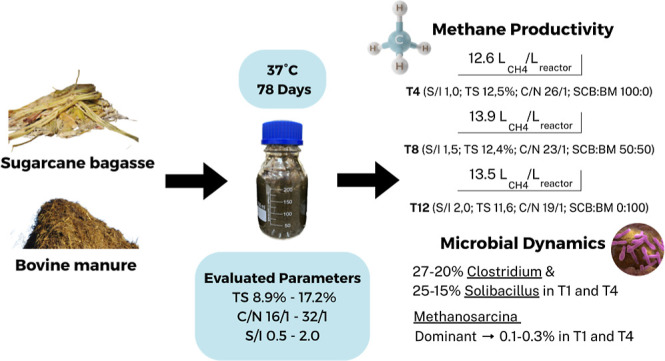

## 1 Introduction

The generation of animal manure and agro-industrial waste is a growing global concern due to its associated potential environmental impacts and the use of natural resources, such as water, nutrients, and energy. In agribusiness, Brazil stands out as the world’s largest producer of sugar cane, with more than 768 million tons per year, followed by India, China, and Thailand with annual production of 348, 123, and 87 tons per year, respectively.^1^ Sugar cane bagasse (SCB) is the primary waste generated in the sugar and ethanol industries, with an estimated 250 kg of bagasse produced for every ton of processed plant material.^2^ This results in the annual generation of approximately 192 million tons of bagasse in Brazil alone in 2023. Due to excessive production, only 50% of bagasse is utilized to meet energy needs; however, burning bagasse contributes to the emission of greenhouse gases (GHG) and exacerbates climate change.^3^

In the livestock scenario, Brazil also stands out in dairy cattle farming with 15.7 million dairy cows and an annual production of approximately 33 billion liters, only behind the European Union, India, the United States, and China.^4–6^ In the dairy production sector, the primary sources of GHG emissions include animal feed production, enteric fermentation, and waste generation, with the latter accounting for up to 30% of total emissions. The significant contribution of manure to gas emissions is due to the microbial decomposition of organic matter and the direct release of methane by the cattle themselves during the digestive process, intensifying the environmental impact of dairy farming.^7,8^

Anaerobic digestion (AD) is a sustainable and efficient solution to mitigate these impacts, promoting the valorization of waste through the degradation of organic matter by a set of microorganisms in the absence of oxygen.^9^ The process occurs in four main steps (hydrolysis, acidogenesis, acetogenesis, and methanogenesis), with hydrolysis being a limiting step during the anaerobic degradation of lignin-rich residues such as bagasse in comparison to traditional substrates such as animal manure, since it involves limited initial degradation of the lignocellulose.^10,11^ The efficiency of the AD process depends primarily on factors such as temperature, carbon-to-nitrogen ratio (C/N), substrate-to-inoculum ratio (S/I), and the microbiota composition of the system.^12^

The biogas resulting from the AD process stands out as a renewable source of energy capable of diversifying the energy matrix, reducing carbon emissions and adding value to organic waste within the circular economy. Biogas production represents a promising and sustainable alternative, bringing economic, environmental, and social benefits by transforming environmental liabilities into an efficient source of energy.^13^ Biogas is predominantly composed of methane (50–60%) and carbon dioxide (30–40%), being used for the generation of thermal and electrical energy, and when subjected to purification and impurity removal, it can achieve performance comparable to compressed natural gas (CNG) used for vehicular mobility.^14^

The efficient conversion of substrates with high total solids (TS) content, such as SCB and bovine manure, remains a significant global challenge.^15,16^ Solid-state anaerobic digestion (SS-AD) emerges as an alternative for treating these wastes, as it operates with a lower volume of water and a higher solid content in the reactor. The strategy offers higher volumetric methane yields, lower water demands, and reduced energy consumption in the process, as well as smaller reactor sizes and easier handling of byproducts.^17^ However, SS-AD has some disadvantages, such as difficulty in mass transfer and high organic load, tending to the accumulation of inhibitors such as ammonia and volatile fatty acids, which can limit the efficiency of the process.^18^

Zhang et al. evaluated the increase in methane production from distiller’s grains and cattle manure in SS-AD.^19^ The highest biogas yield was 315 L kg_VS_^–1^ from the codigestion of grains with bovine manure in the proportion of 70:30, an S/I ratio of 1.0, and 15% TS. Arelli et al. investigated the potential impact of cellulose-degrading bacteria that could be bioaugmented on the AD of SCB to increase methane yield.^20^ The maximum methane yield of 440 L kg_VS_^–1^ was obtained with the bioaugmented bagasse with 40% TS, while the methane yield of the bagasse that did not undergo the addition of bacterial strains was only 340 L kg_SV_^–1^, confirming the increase in methane production resulting from bioaugmentation. On the other hand, Liew, Shi, and Li evaluated the SS-AD of lignocellulosic biomass, and the highest accumulated methane production was related to corn straw with only 81 L kg_SV_^–1^ operating with S/I of 2.0 and 22% TS.^21^ These results show that there is a wide variety of operational parameters, requiring optimized values for each type of substrate, as well as the investigation of the relationship between these operational parameters and microbiology.^16^

Monodigestion of these residues may be ineffective due to the initial limiting step of anaerobic digestion, which requires an equilibrated moisture content, a balanced nutrient ratio such as C/N, and the availability of easily degradable organic matter to support the growth of microorganisms.^22^ The scientific literature reports ideal C/N for AD from 20/1 to 30/1,^23^ but lignocellulosic substrates in AD often need adjustments to optimize biogas production, with C/N values below 20/1.^24^ Similarly, adjusting the S/I ratio is crucial in aligning the organic load with the degradation capacity of the microorganisms, as well as the high proportion of solids that can also compromise nutrient diffusion and microbial activity, thereby reducing the overall process efficiency.^25^

Despite the growing interest in SS-AD through empirical evaluation of process performance, studies integrating operational optimization with microbial community dynamics remain insufficiently explored, particularly for lignocellulosic substrates. Therefore, this study aimed to evaluate and optimize the codigestion of SCB and BM under different S/I and C/N ratios, moisture contents, and organic loads in batch assays by combining physicochemical and microbiological assessments to identify operational conditions that maximize methane yield while maintaining process stability. This integrated approach moves beyond descriptive optimization and contributes to a deeper understanding of the stability and resilience in SS-AD systems.

## 2 Materials and Methods

### 2.1 Inoculum and Substrates

The experiments were carried out with a mixture of two inoculum sources: a liquid digestate from a mesophilic pilot-scale reactor (LD) collected at the International Center on Renewable Energy and Biogas, CIBiogás, treating animal manure, and the solid fraction of a digestate (SD) collected in a mesophilic real-scale reactor at the Itaipu Demonstration Unit treating food waste in Foz do Iguaçu/Brazil. The two inocula were mixed in a ratio of 0.5:1 (w/w). After homogenization, the inoculum was incubated at 37 °C for 7 days to facilitate degassing. The mixture between the digestates was carried out to increase the % TS content in the reactors and meet the SS-AD parameters. The substrates used in the experiments consisted of bovine manure (BM) from a dairy farm that raises cattle on pasture and sugar cane bagasse (SCB) from a small sugar cane juice producer, both located in Medianeira, Brazil. The SCB was washed with tap water to remove excess sugars to simulate the bagasse generated in sugar and ethanol industries and then air-dried for 3 days, followed by overnight oven drying at 60 °C and crushed up to 2 cm in particle size. The BM and SCB were frozen in airtight plastic bags at −20 °C until further use.

### 2.2 Biochemical Methane Potential (BMP) Assay

The BMP bioassays were performed using glass flasks with a volume of 250 mL (50% headspace) with butyl rubber septa and screw caps. The treatments were performed in triplicate in addition to assays containing only inoculum (negative control) and a mixture of inoculum with microcrystalline cellulose (positive control) to evaluate the endogenous production and the quality of the inoculum (eq 1).^26^

1 $$BMP=\frac{V_{1}-\left[V_{2}\left(M_{1}\times {VS}_{IN}\right)\right]}{\left(M_{2}\times {VS}_{SUB}\right)}\times \%\;{CH}_{4}$$

where BMP is biochemical methane potential (L_CH_4__ kg_SV_^–1^ added); *V*_1_: total volume of biogas in the reactor containing inoculum and substrate (L); *V*_2_: specific volume of reactor biogas containing only inoculum (L kg_SV_^–1^); M_1_: added inoculum mass (kg); *M*_2_: mass of substrate added (kg); VS_IN_: volatile solid content of the inoculum (%); VS_SUB_: volatile solid content of the substrate (%); and % CH_4_: methane concentration in the measured biogas (%).

Nitrogen gas (N_2_) was recirculated to degas the reactors, instantly turning the environment anaerobic. Subsequently, the flasks were sealed and kept in an oven at 37 °C with no agitation.^27^ During the 78 day incubation period, biogas production was monitored daily for the first 10 days, every 2 days until the 20th day, and every 3 days thereafter until the experiment concluded. Biogas volume was measured using 20 and 100 mL glass syringes. The biogas and methane yields were corrected according to the Standard Temperature and Pressure (STP) conditions (273.15 K and 1013 mbar) and expressed in NL kg of SV-1 added (eq 2).

2 $$V_{0}=V\times \frac{\left(P_{L}-P_{w}\right)T_{0}}{P_{0}\times T}$$

where *V*_0_ is the normal volume of biogas (mL_N_); *V*: volume of biogas recorded (mL); *P*_L_: atmospheric pressure on the day of measurement (mbar); *P*_w_: water vapor pressure (1013 mbar); and *T*: incubator temperature (37 °C).

The water vapor pressure inside the reactors was determined based on the incubator temperature using eq 3.^28^

3 $$P_{w}=10^{81,962-\frac{1.730,63}{T-39,724}}$$

The experimental design involved varying the S/I ratio between 0.5 and 2.0, as well as the proportion of SCB/BM, from 100:0, 50:50, and 0:100, as presented in Table S1 of Supporting Information. The TS content ranged from 8.9 to 17.2%, while the C/N ratio ranged from 16/1 to 37/1 as a result of the substrates’ mixing ratios. The organic matter concentration in the reactors varied in the range of 67.0 to 89.1 g_VS_ L^–1^, while Zhang et al. found an organic loading of 60 g_VS_ L^–1^ for codigestion of sweet potato vine and dairy manure in semidry conditions.^29^

### 2.3 Analytical Methods

The determination of pH was carried out according to the Standard Methods^30^ using the 4500 H+ method, as well as total solids (TS) and volatile solids (VS), which were determined using the 2540 G method. The total Kjeldahl nitrogen (TKN) was analyzed using the micro-Kjeldahl method,^31^ and the C/N ratio was based on the total organic carbon (TOC) ratio, obtained by dividing VS on a dry basis by the factor of 1.8, by the percentage of TKN in the sample.^32^

Total alkalinity (TA) and the relationship between intermediate and partial alkalinity (IA/PA) were determined by the potentiometric method.^33^ For this, 20 g of the sample was diluted in 80 g of distilled water, followed by stirring for 15 min and centrifugation for 20 min to perform the analysis with the supernatant fraction. After the sample was prepared, with the aid of a pH meter (Instrutherm/pH-5000), a 0.1 N sulfuric acid solution (H2SO4) was used to lower the pH to the stopping points of 5.75 and 4.3. The determination of PA was performed according to the calculation presented by eq 4. After the PA and IA were determined, the IA/PA ratio was calculated as the ratio between these values. Finally, the TA was determined by summing the PA and IA.

4 $$\left(\frac{Volume\;of\;titrant\;up\;to\;pH\;5.75\;\left(mL\right)\times acid\;concentration\;\left(\frac{mol}{L}\right)}{Volume\;of\;sample\;\left(mL\right)}\right)\times 100.000$$

To determine the IA, the same calculation was performed as for the PA, considering only the volume of titrant solution used up to a pH of 4.3, as shown in eq 5.

5 $$\left(\frac{Volume\;of\;titrant\;up\;to\;pH\;4.30\;\left(mL\right)\times acid\;concentration\;\left(\frac{mol}{L}\right)}{Volume\;of\;sample\;\left(mL\right)}\right)\times 100.000$$

During the BMP, the contents of methane (CH_4_) and carbon dioxide (CO_2_) were monitored using gas chromatography^34^ on a PerkinElmer Clarus 680 instrument. The chromatograph is equipped with a Thermal Conductivity Detector (TCD) and a 30 m long, 0.32 mm internal diameter Plot Q packaged column. Helium gas is used as a carrier gas with a flow rate of 30 mL min^–1^,^,^ and the furnace of the equipment is programmed to reach 200 °C at a rate of 10 °C min^–1^.

The quantification of volatile fatty acids (VFAs) (including formic, acetic, propionic, butyric, isobutyric, valeric, and isovaleric acids) was performed using high-performance liquid chromatography (HPLC). For this analysis, a Biorad Aminex HPX-87H column (300 × 7.8 mm) was used, operating with a mobile phase consisting of 0.05 M sulfuric acid solution at a constant flow rate of 0.60 mL min^–1^. Compound detection was carried out using a UV detector.

### 2.4 Characterization of the Microbial Community

The analysis of the microbial community was carried out for samples T1 and T4. A 50 mL volume of sample was collected and centrifuged for 10 min at 5000 rpm. The supernatant was discarded, and the solid fraction (microbial biomass) was stored at −80 °C for sample preservation. DNA extraction was performed using the DNeasy Power Soil kit (Qiagen) according to the manufacturer’s instructions. The Qubit fluorometer (Thermo Fisher Scientific) was used to quantify the genetic material. Microbial characterization was performed through next-generation sequencing on the Illumina MiSeq Platform (Illumina Inc., San Diego, CA, USA). Library preparation was conducted by PCR amplification using primers 341F/805R, which target the V3–V4 variable region of the 16S rRNA gene for both bacteria and archaea.^35,36^

The quality of the sequenced nucleotides was assessed with FastQC.^37^ The resulting reads were imported, processed, and analyzed using the QIIME 2 pipeline (Quantitative Insights Into Microbial Ecology).^38^ Sequences of primers and adapters were removed by using the q2-cutadapt plugin. Chimeras, noise, and other sequencing errors were also removed by the DADA2 algorithm (Divisive Amplicon Denoising Algorithm). Singletons obtained during the process were excluded by using the q2-feature-table plugin. Amplicon sequence variants, their respective FASTA sequences, and relative abundance values were used for taxonomic profiling using the feature-classifier plugin, with the classify-sklearn method, to compare sequences against the SILVA database (v. 138).^39^

### 2.5 Statistical Analysis

The replicates of each treatment were subjected to analysis of variance (ANOVA) to determine whether there were significant differences between the means. To group the BMP data and perform the mean comparison test, the Fisher test was applied with a significant level (α) of 5% using Minitab 16.

## 3 Results and Discussion

### 3.1 Characteristics of the Inoculum and Substrates

The pH of the mixed inoculum was 7.4 (Table 1). The total alkalinity of 3150 mg L^–1^ CaCO_3_ and an IA/PA ratio of 0.24 were slightly higher than the minimum recommended by the literature, such as Holliger et al., suggesting 3000 mg L^–1^ CaCO_3_,^27^ and Ripley, Boyle, and Converse recommending an IA/PA of inoculum higher than 0.30.^33^ The TS and VS/TS of the mixed inoculum were 7.2% and 80.8%, while the C/N was 10/1, within the ideal range according to Zhu et al., between 10/1 and 15/1.^24^

**Table 1:** Physicochemical Parameters of the Inoculum and Substrates^a^

sample	pH	TA (mg L^–1^ CaCO_3_)	IA/PA	TS (%)	VS (%)	VS/TS (%)	TKN (% ST)	C/N
liquid digestate	8.1	5775	0.04	4.6 ± 0.0	2.8 ± 0.0	61.3	8.5 ± 0.1	4/1
solid digestate	7.3	2250	0.50	8.4 ± 0.5	7.5 ± 0.4	89.4	2.6 ± 2.6	19/1
INO_mixed_	7.4	3150	0.24	7.2 ± 0.1	5.8 ± 0.1	80.8	4.6 ± 0.0	10/1
SCB	N.D.	N.D.	N.D.	96.3 ± 0.3	94.7 ± 0.2	98.3	0.2 ± 0.0	227/1
BM	7.0	1625	0.86	16.1 ± 0.2	12.2 ± 0.1	75.5	1.8 ± 1.8	23/1

a SCB: sugar cane bagasse; BM: bovine manure; TA: total alkalinity; IA/PA: intermediate alkalinity/partial alkalinity ratio; TS: total solids; VS: volatile solids; VS/TS: volatile solids/total solids ratio; TKN: total Kjeldahl nitrogen; C/N: carbon/nitrogen ratio; N.D.: not determined.

The TS content of SCB was 96.3%, reflecting the substrate drying process applied to minimize heterogeneity in the BMP assays. The VS/TS ratio of 98.3% from SCB suggests a considerable amount of organic matter, showing the potential of this substrate for biogas production. However, the C/N of the SCB was 227/1, indicating too low biodegradability of its organic matter for anaerobic monodigestion. The TS and VS/TS of the BM used as the cosubstrate were 16.1 and 75.5%, indicating a high proportion of organic matter with potential for conversion into biogas, confirmed by its C/N of 23/1. The pH and total alkalinity of the BM were 7.0 and 1625 mg L^–1^ CaCO_3_, indicating its potential to contribute to the buffer effect during the SS-AD.

### 3.2 Methane Yield Optimization through C/N, S/I, and Substrate Mixing Ratios

The BMP assays lasted 78 days, a slightly longer period than the 60 days reported by Wang et al. using cucumber, corn stage, and pig manure as substrates^40^ and Paranhos et al. using poultry litter and lignocellulosic biomass.^41^ Overall, the results indicate that methane production in SS-AD of SCB and BM is constrained by a narrow operational window defined by TS content, S/I ratio, and C/N balance. Excessive organic loading and high TS imposed physical and metabolic limitations that reduced process efficiency and stability, indicating that SS-AD performance is governed by a couple of physical–biological constraints.

The highest daily biogas rate occurred during the first 10 days of digestion in treatments T1 (S/I 0.5, TS 10%, C/N 21/1, SCB/BM 100:0) and T7 (S/I 1.5, TS 14.9%, C/N 32/1, SCB/BM 100:0) with a maximum biogas rate of 16 L kg_SV_^–1^ d^–1^ (Figure 1). The early peak may be attributed to the presence of residual sugar in the SCB, which is readily degradable and can accelerate initial biogas production. Similar values ranging from 16.2 to 17.0 L kg_SV_^–1^ d^–1^ were reported by Alsebiey et al.^42^ for the anaerobic codigestion of SCB and BM at the proportion of 70:30 (m/m). On the other hand, the lowest biogas production rates were recorded in the treatments T10 (S/I 2.0, TS 17.2%, C/N 37/1, SCB/BM 100:0), T11 (S/I 2.0, TS 13.4%, C/N 25/1, SCB/BM 50:50), and T12 (S/I 2.0, TS 11.6%, C/N 19/1, SCB/BM 0:100), ranging between only 4 and 6 L kg_SV_^–1^ d^–1^, observed between the second and seventh day of digestion (Figure 1). This behavior may be associated with the higher TS (11.6–17.2%), organic load (organic matter >78.5 g_VS_ L^–1^), S/I of 2.0, and C/N (19/1–37/1), which influenced the availability of nutrients to the anaerobic microbiota, regardless of the proportion between the substrates (SCB/BM). Under such conditions, operational limitations in anaerobic digestion systems are frequently linked to reduced contact between microorganisms and substrates, particularly at an elevated TS content. In this context, TS is widely used as an indicator of digester performance and efficiency.^43^

**Figure 1:**
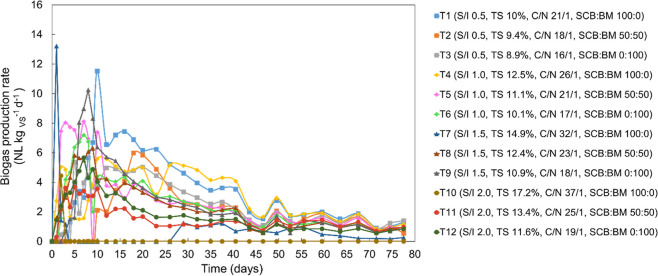
Daily biogas production from treatments performed for S/I of 0.5, 1.0, 1.5, and 2.0.

The treatments T1 (S/I 0.5, TS 10%, C/N 21/1, SCB/BM 100:0), T3 (S/I 0.5, TS 8.9%, C/N 16/1, SCB/BM 0:100), T4 (S/I 1.0, TS 12.5%, C/N 26/1, SCB/BM 100:0), T5 (S/I 1.0, TS 11.1%, C/N 21/1, SCB/BM 50:50), and T8 (S/I 1.5, TS 12.4%, C/N 23/1, SCB/BM 50:50) resulted in the statistically highest biogas yields (*p* < 0.05) of 442, 358, 390, and 371 L kgSV^–1^ (Figure 2). Shobaju et al.^44^ evaluated the anaerobic codigestion under wet conditions (5% TS) also using SCB and BM at the proportion of 80:20 (m/m) and reported lower values of 225 L_CH_4__ kg_VS_^–1^ when compared to this study, evidencing that the optimized increase in the TS and organic load can improve methane yields of these substrates. On the other hand, the biogas yield from treatments T7 (S/I 1.5, TS 14.9%, C/N 32/1, SCB/BM 100:0) and T11 (S/I 2.0, TS 13.4%, C/N 25/1, SCB/BM 50:50) resulted in the statistically lowest values (*p* < 0.05) of 190 and 187 L kg_SV_^–1^ (Figure 2), while no biogas was detected from T10 (S/I 2.0, TS 17.2%, C/N 37/1, SCB/BM 100:0), suggesting a possible failure in the microbial inhibition process caused by the higher TS (17.2%) and C/N (37/1). These results evidence the negative effect on the SS-AD when operational parameters are increased above the optimized range.

**Figure 2:**
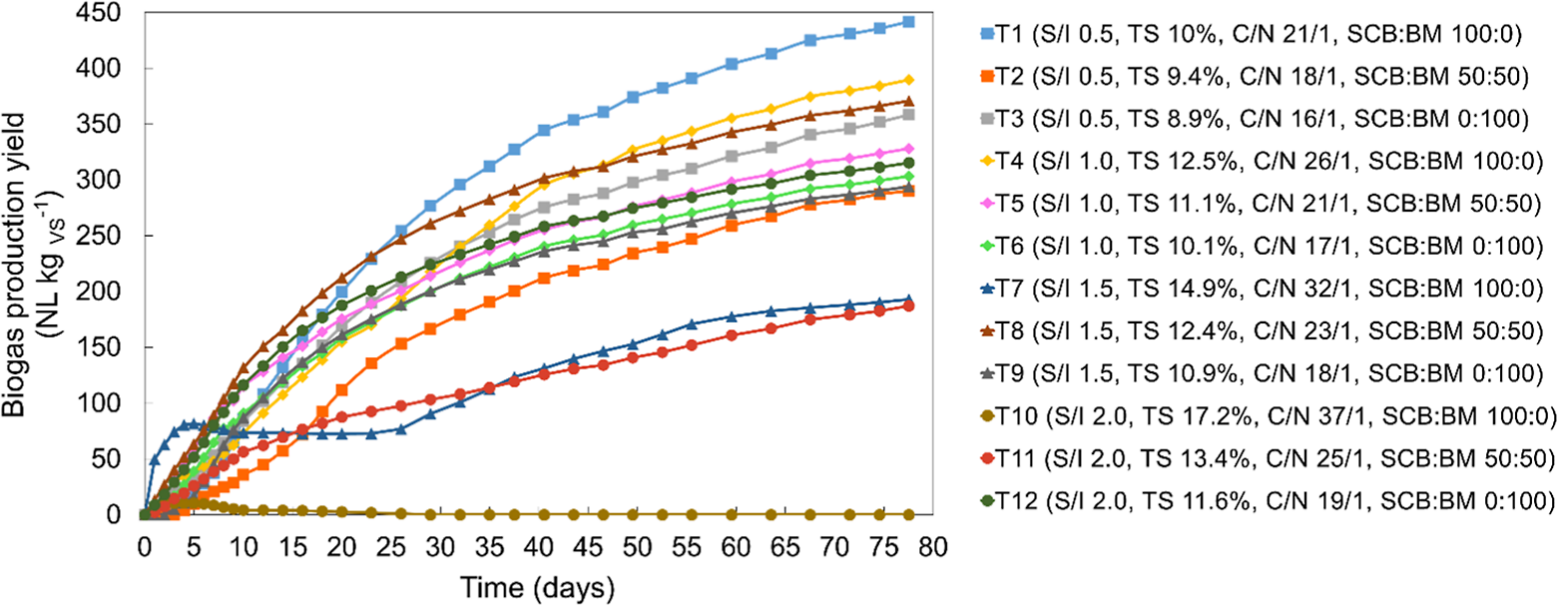
Cumulative biogas production from treatments performed for S/I of 0.5, 1.0, 1.5, and 2.0.

The methane content in the biogas varied between 33% and 62% (Table 2), which is consistent with the values reported in the literature for the codigestion of distillers’ grains and cattle manure, where methane contents generally ranged from 50% to 70%^19^ with the highest values observed from T1 (S/I 0.5, TS 10%, C/N 21/1, SCB/BM 100:0) and T4 (S/I 1.0, TS12.5%, C/N 26/1, SCB/BM100:0), indicating greater energy potential from the substrates when S/I, C/N, and O.M. concentration are optimized. On the other hand, T7 showed a very low methane content of 33%, which was significantly lower than the average 55% obtained from the other treatments, suggesting limited methanogenic metabolism due to the higher TS (14.9%) and C/N (32:1) conditions.

**Table 2:** Biochemical Biogas and Methane Potential and Volumetric Efficiency of the Treatments^a^

	biochemical methane potential		methane productivity			
treatment	(NL_CH___4__kg_VS_^–1^)	(NL_CH___4__kg_residue_^–1^)	(L_CH___4__/L_reactor_)	CH_4_ (%)	*T*_80_ (d)	*T*_90_ (d)
T1	267 ± 9^A^	254 ± 9^A^	9.7 ± 0^DE^	60 ± 0	44	58
T2	173 ± 30^CD^	37 ± 6^C^	6.8 ± 2^F^	59 ± 0	46	61
T3	209 ± 43^BC^	25 ± 5^D^	8.4 ± 1^EF^	58 ± 0	46	61
T4	246 ± 6^AB^	233 ± 6^A^	12.6 ± 0^AB^	62 ± 2	46	58
T5	213 ± 0^B^	45 ± 0^BC^	11.7 ± 0^BC^	57 ± 0	41	57
T6	173 ± 17^CD^	21 ± 2^D^	10.2 ± 1^CD^	57 ± 1	42	57
T7	63 ± 0^F^	60 ± 0^B^	4.3 ± 0^G^	33 ± 0	51	58
T8	21C2 ± 0^B^	44 ± 0^BC^	13.9 ± 0^A^	57 ± 0	40	57
T9	163 ± 14^D^	20 ± 2^D^	11.1 ± 1^BCD^	55 ± 1	41	57
T10	0^G^	0^E^	0^H^	0	0	0
T11	105 ± 21^E^	22 ± 4^D^	8.3 ± 1^EF^	56 ± 0	65	65
T12	169 ± 0^D^	21 ± 0^D^	13.5 ± 0^A^	54 ± 0	56	56

a Statistical analysis using Fisher’s test; equal letters indicate that there is no significant difference with α = 5% probability by Fisher’s test.

In terms of methane yield, the statistically most efficient conditions (*p* < 0.05) were observed for the treatments T1 (S/I 0.5, TS 10%, C/N 21, SCB/BM 100:0) and T4 (S/I 1.0, TS 12.5%, C/N 26/1, SCB/BM 100:0), reaching 267 and 246 NL_CH_4__ kg_VS_^–1^, respectively. In addition, the same treatments (T1 and T4) also stood out when methane yield was expressed based on the mass of waste added, reaching statistically similar values (*p* < 0.05) of 254 and 233 NL kg_residue_^–1^, respectively, demonstrating that the balanced proportion between the substrate and inoculum, combined with a moderate C/N (21/1–26/1), favored the conversion into biogas. Similarly, Ahmad et al. found an optimized C/N ratio of 20/1–25/1 for the codigestion of municipal solid waste and food waste.^45^

The *T*_80_ and *T*_90_ parameters were calculated based on the linear prediction of 80 and 90% of the cumulative BMP values, respectively, that ranged from 40 to 65 days (T80) and 56 to 65 days (T90) across all treatments. Edwiges and Kaparaju reported similar *T*_90_ values, ranging from 59 to 66 days, for the dry digestion of SCB,^17^ indicating a relatively higher degradation time for the SS-AD compared to traditional wet-AD due to the hydrolysis-limited step (Figure 2).

The highest volumetric methane yields (NL_CH_4__/L_reactor_), with “reactor” being the operational volume (a mixture of substrate and inoculum), were detected in treatments T4 (S/I 1.0; TS 12.5%; C/N 26/1; SCB/BM 100:0), T8 (S/I 1.5; TS 12.4%; C/N 23/1; SCB/BM 50:50), and T12 (S/I 2.0; TS 11.6%; C/N 19/1; SCB/BM 0:100), with statistically similar results (*p* < 0.05) of 12.6, 13.9, and 13.5 NL_CH_4__/L_reactor_, respectively. The range of TS in which the highest volumetric methane yields were observed was 11.6% to 12.5%, while C/N ranged from 19/1 to 26/1.

On the other hand, treatment T7 resulted in the lowest volumetric productivity of only 4.3 NL_CH_4__/L_reactor_. This treatment was performed with an S/I of 1.5, a TS of 14.9%, a C/N of 32/1, and an SCB of 100%. The TS and C/N were higher in this treatment than the optimized average of the T4, T8, and T12 (approximately 12% TS and C/N between 19/1 and 26/1), showing that the progression of organic load in monodigestion of carbon-rich and nitrogen-poor residues results in operational problems and significant yield loss. This can also be observed from the perspective of organic matter concentration since the treatments with the highest methane productivity were operated with 72.1 to 89.1 g_VS_ L^–1^, while the lowest results were observed with a concentration of 75.9 g_VS_ L^–1^; that is, the quality of the organic matter inside the reactor in a solid state, observed by the C/N, plays a crucial role when compared to S/I and TS content. These findings highlight that in SS-AD systems, the C/N ratio directly influences microbial balance and methane yield, as both excessive nitrogen and insufficient carbon can lead to ammonia inhibition or substrate limitation, respectively, ultimately affecting biogas productivity.^14^

Regarding substrate composition, both the monodigestion of SCB at an S/I of 1.0 and the codigestion of SCB/BM at 50:50 and the monodigestion of BM at an S/I of 2.0 showed higher volumetric methane productivity, indicating that the monodigestion of the lignocellulosic-rich substrate (SCB) promotes adequate volumetric methane when S/I is limited to 1/1, while an S/I higher than 1/1 requires a nitrogen-rich substrate (BM).^40^

The correlation between the experimental conditions and the BMP values resulted in strong negative linear coefficients (*p* < 0.05) of −0.786 with TS and −0.704 with C/N (Table 3). Such values indicate that the increase in solid concentration and the proportion between carbon and nitrogen can potentially compromise the efficiency of anaerobic digestion under solid-state conditions. The results suggest that high TS content may hinder the degradation of organic matter, possibly due to limitations in nutrient transport and diffusion of substrates to methanogenic microorganisms.^25^ Similarly, a high C/N may indicate excess available carbon without an adequate amount of nitrogen to sustain microbial metabolism, compromising methane production.

**Table 3:** Correlation between the Parameters and the Response Variables of the BMP Test

parameter	S/I	TS (%)	C/N
TS (%)	0.748^a^		
C/N	0.529^a^	0.958^a^	
BMP (L kg_SV_^–1^)	–0.669^a^	**–0.786^a^**	**–0.704^a^**
methane productivity (L_methane_/L_reactor_)	–0.139	–0.562	–0.650^a^
BMP (L kg_MN_^–1^)	–0.456	–0.155	–0.034

a *p*-Value less than the significance level of 0.05.

The coefficient of determination (*R* ^2^) obtained between BMP and TS data was 0.7238, indicating that the TS content can explain approximately 72.38% of the variation in methane yield (Figure 3a). The graph curve indicates that treatments with a TS content greater than 12.5% had a progressive reduction in the methane yield, with a sharp drop to values above 14%.

**Figure 3:**
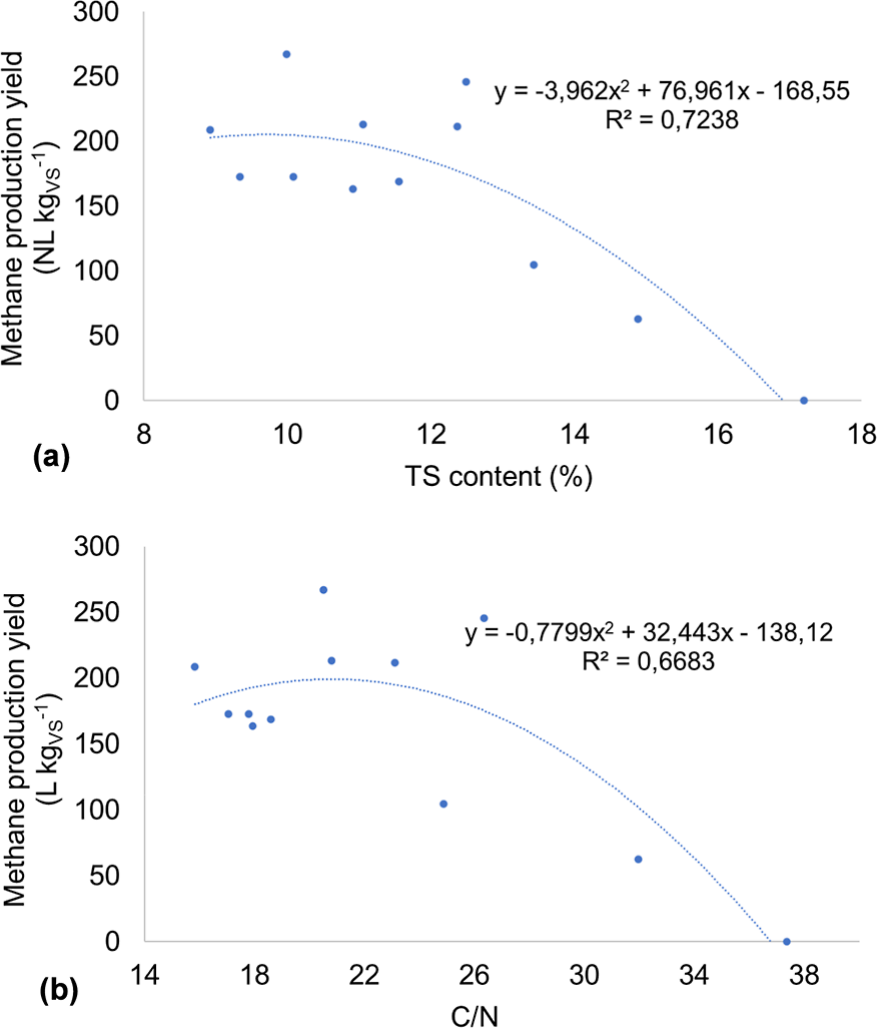
Correlation between BMP and TS content (a) and the C/N ratio (b).

In addition, the correlation between the BMP and C/N resulted in an R^2^ of 0.6683, indicating that 66.83% of the variability in the methane yield can be explained by the C/N ratio (Figure 3b). Although lower than the first model, this value still suggests a significant influence of this variable on methane production, confirming that for the experiment, C/N values above 26/1 compromise the process efficiency.

### 3.3 Characteristics of the Digestates after the SS-AD at Different Operational Conditions

The concentrations of the organic matter in the digestates after SS-AD indicate the degree of biodegradation of the organic compounds. The SS-AD batch assays started with TS content between 7.2% and 17.2% and ended with TS between 5.2% and 11.4% (Table 4). The average 26% reduction in the TS content can be attributed to biochemical conversions and physicochemical changes in the solid–liquid balance of the system, such as the solubilization of organic matter by hydrolytic and acidogenic bacteria.

**Table 4:** Physicochemical Parameters of Digestate before and after the BMP Assay^a^

treatments	TS (%) day 1	TS (%) day 76	VS (%) day 1	VS (%) day 76	C/N day 1	C/N day 76	TAN (%_ST_) day 76	FA (mg kg_residue_^–1^) day 76	pH day 76	TA (g L^–1^ CaCO_3_) day 76	IA/PA day 76
INO	7.2 ± 0.0	5.2 ± 0.3	5.8 ± 0.0	4.1 ± 0.3	14/1	7/1	4.1 ± 0.5	621	8.5	4.5	0.13
T1	10.0 ± 0.0	6.9 ± 0.2	8.6 ± 0.0	5.5 ± 0.2	21/1	6/1	4.1 ± 0.0	109	7.5	4.0	0.23
T2	9.4 ± 0.0	6.7 ± 0.3	7.7 ± 0.0	5.2 ± 0.3	18/1	5/1	4.0 ± 0.1	201	7.8	3.9	0.15
T3	9.0 ± 0.0	6.6 ± 0.5	7.1 ± 0.0	4.9 ± 0.4	16/1	8/1	2.4 ± 0.2	890	9.0	4.2	0.14
T4	13.0 ± 0.0	8.7 ± 0.4	11.0 ± 0.0	7.2 ± 0.3	26/1	5/1	3.2 ± 0.1	257	7.9	3.7	0.10
T5	11.1 ± 0.0	7.7 ± 0.2	9.1 ± 0.0	5.9 ± 0.1	21/1	6/1	2.9 ± 0.0	644	8.5	4.0	0.14
T6	10.1 ± 0.0	7.1 ± 0.1	7.9 ± 0.0	5.2 ± 0.1	17/1	5/1	3.1 ± 0.0	635	8.5	3.5	0.05
T7	15.0 ± 0.0	11.4 ± 0.7	13.3 ± 0.0	9.9 ± 0.6	32/1	5/1	3.4 ± 0.2	25	6.7	1.8	1.80
T8	12.4 ± 0.0	8.7 ± 0.3	10.3 ± 0.0	6.8 ± 0.3	23/1	6/1	3.2 ± 0.1	387	8.1	3.5	0.11
T9	10.9 ± 0.0	7.9 ± 0.1	8.5 ± 0.0	5.7 ± 0.0	18/1	5/1	3.0 ± 0.2	329	8.1	3.7	0.22
T10	17.2 ± 0.0	15.6 ± 1.6	15.6 ± 0.0	13.9 ± 1.4	37/1	6/1	4.8 ± 0.1	0	4.3	-	-
T11	13.4 ± 0.0	9.3 ± 0.5	11.1 ± 0.0	7.0 ± 0.5	25/1	5/1	3.0 ± 0.2	680	8.4	3.3	0.18
T12	11.6 ± 0.0	8.2 ± 0.3	8.9 ± 0.0	5.7 ± 0.3	19/1	5/1	2.9 ± 0.1	270	8.0	2.5	0.11

a TS: total solids; VS: volatile solids; C/N: carbon/nitrogen ratio; TAN: total ammonia nitrogen; FA: free ammonia; TA: total alkalinity; IA/PA: intermediate alkalinity/partial alkalinity ratio.

VS removal varied between 10.9% and 36.9% for all treatments, reflecting the different degrees of substrate biodegradability and operational conditions. The VS reduction was around 36% in the treatments T1 (S/I 0.5; TS 10.0%; C/N 21/1; SCB/BM 100:0), T11 (S/I 2.0; TS 13.4%; C/N 25/1; SCB/BM 50:50), and T12 (S/I 2.0; TS 11.6%; C/N 19/1; SCB/BM 0:100) (Figure 4), suggesting greater degradation of organic matter for these treatments. This value is notably higher than the 17% VS removal reported for the anaerobic codigestion of lignocellulosic and lipidic wastes with cattle manure under mesophilic conditions, indicating greater degradation efficiency in the present study.^46^ In contrast, VS removal from T10 (S/I 2.0; TS 17.2%; C/N 37/1; SCB/BM 100:0) was only 11%, showing a limited anaerobic degradation due to the organic matter overload, especially the lignocellulosic biomass from the SCB.

**Figure 4:**
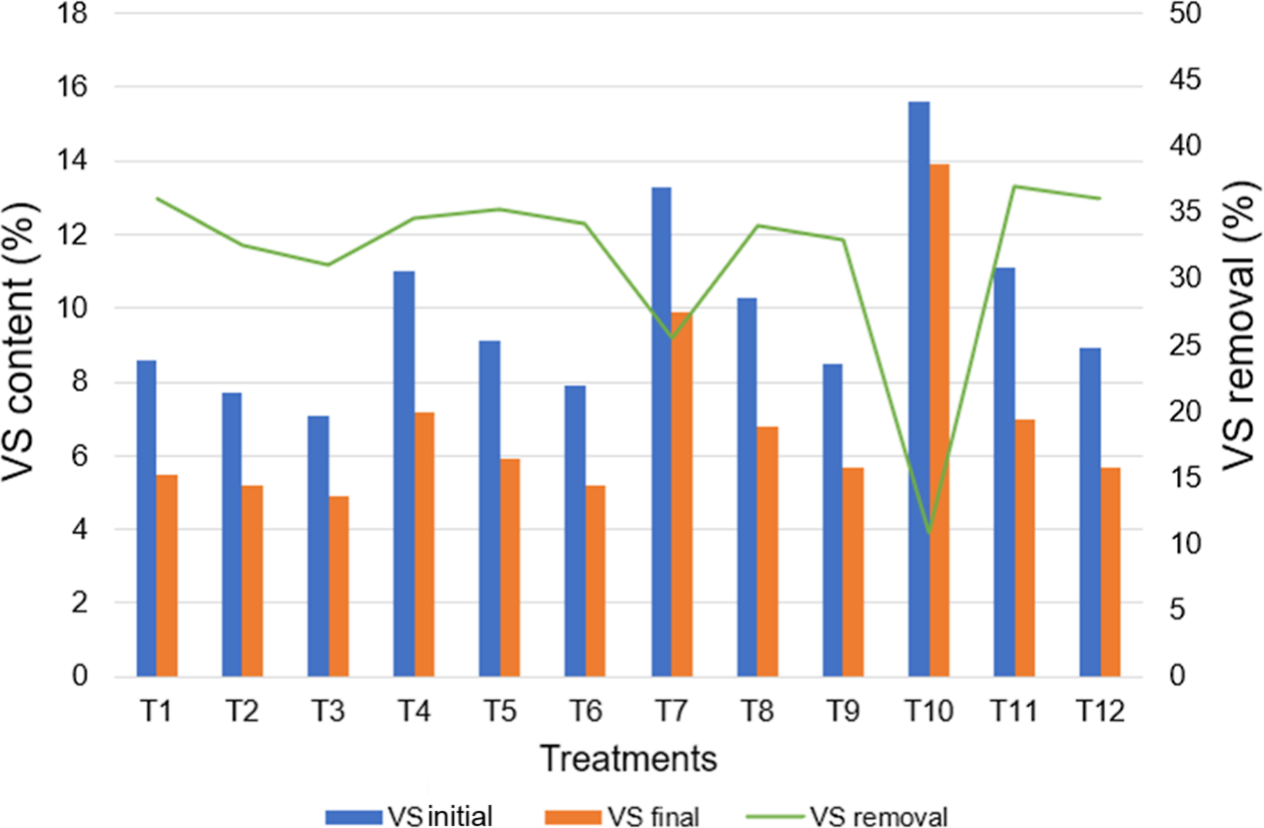
SV content at the beginning and end of the experiment and SV removal.

The C/N ratio also had significant variations throughout the tests, ranging from 14/1 to 37/1 at the beginning and from 5/1 to 8/1 at the end. All treatments recorded a reduction in the carbon content, confirming the conversion of carbon into biogas and organic acids. Treatments T4 (S/I 1.0, TS 12.5%, C/N 26/1, SCB/BM 100:0), T7 (S/I 1.5, TS 14.9%, C/N 32/1, SCB/BM 100:0), and T10 (S/I 2.0, TS 17.2%, C/N 37/1, SCB/BM 100:0), which started with higher C/N ratios due to the monodigestion of the SCB, resulted in the highest reduction in terms of C/N (82% average). On the other hand, the T3 (S/I 0.5, TS 8.9%, C/N 16/1, SCB/BM 0:100) resulted in the lower carbon reduction in relation to nitrogen with a final C/N of 8/1 (30% reduction) due to the monodigestion of BM under a much lower organic load (67.0 g_VS_ L^–1^). In addition, it was observed that the monodigestion of the SCB under higher S/I (>1.5), which was the case of T7 (S/I 1.5, TS 14.9%, C/N 32/1, SCB/BM 100:0) and T10 (S/I 2.0, TS 17.2%, C/N 37/1, SCB/BM 100:0), promoted alkalinity consumption with final concentrations under 1800 g CaCO_3_ L^–1^ and IA/PA of 1.80, which were much higher than the 3.000 mg CaCO_3_ L^–1^ and 0.3 reported in the literature for a stable A.D. process.^27^

The total ammonia nitrogen (TAN) in the digestate ranged from 2.4% to 4.8 % TS, and as expected, treatments starting with a balanced C/N, such as T3 (S/I 0.5, TS 8.9%, C/N 16/1, SCB/BM 0:100) and T5 (S/I 1.0, TS 11.1%, C/N 21/1, SCB/BM 50:50), resulted in lower concentrations of TAN, reflecting a more stable environment under the studied conditions. The lowest FA values were observed in treatments T7 (S/I 1.5; TS 14.9%; C/N 32/1; SCB/BM 100:0) and T10 (S/I 2.0; TS 17.2%; C/N 37/1; SCB/BM 100:0), with concentrations under 25 mg kg_residue_^–1^, which were associated with the pH under 6.7 ^47^ and limited or no methanogenic degradation. In contrast, treatments with the statistically higher volumetric methane productivity such as T4 (S/I 1.0; TS 12.5%; C/N 26/1; SCB/BM 100:0), T8 (S/I 1.5; TS 12.4%; C/N 23/1; SCB/BM 50:50), and T12 (S/I 2.0; TS 11.6%; C/N 19/1; SCB/BM 0:100) resulted in equilibrate FA concentrations ranging from 257 to 387 mg kg_residue_^–1^, which were related to their C/N under 26 and pH values between 7.9 and 8.1 and indicated inhibition of bacterial activity when FA is over 400 mg kg_residue_^–1^, as FA concentrations above this threshold can cause more than 50% inhibition of methanogenesis.^10^

Low VFA concentrations were observed in the digestate for almost all treatments, with values up to 221 mg L^–1^ (Figure 5a). The low acid concentrations in the digestate of these treatments indicate a stable and controlled process with suitable operational parameters to support AD without overloading the methanogenic microbiota, as low levels of VFA reflect a balanced and efficient anaerobic digestion.^48^

**Figure 5:**
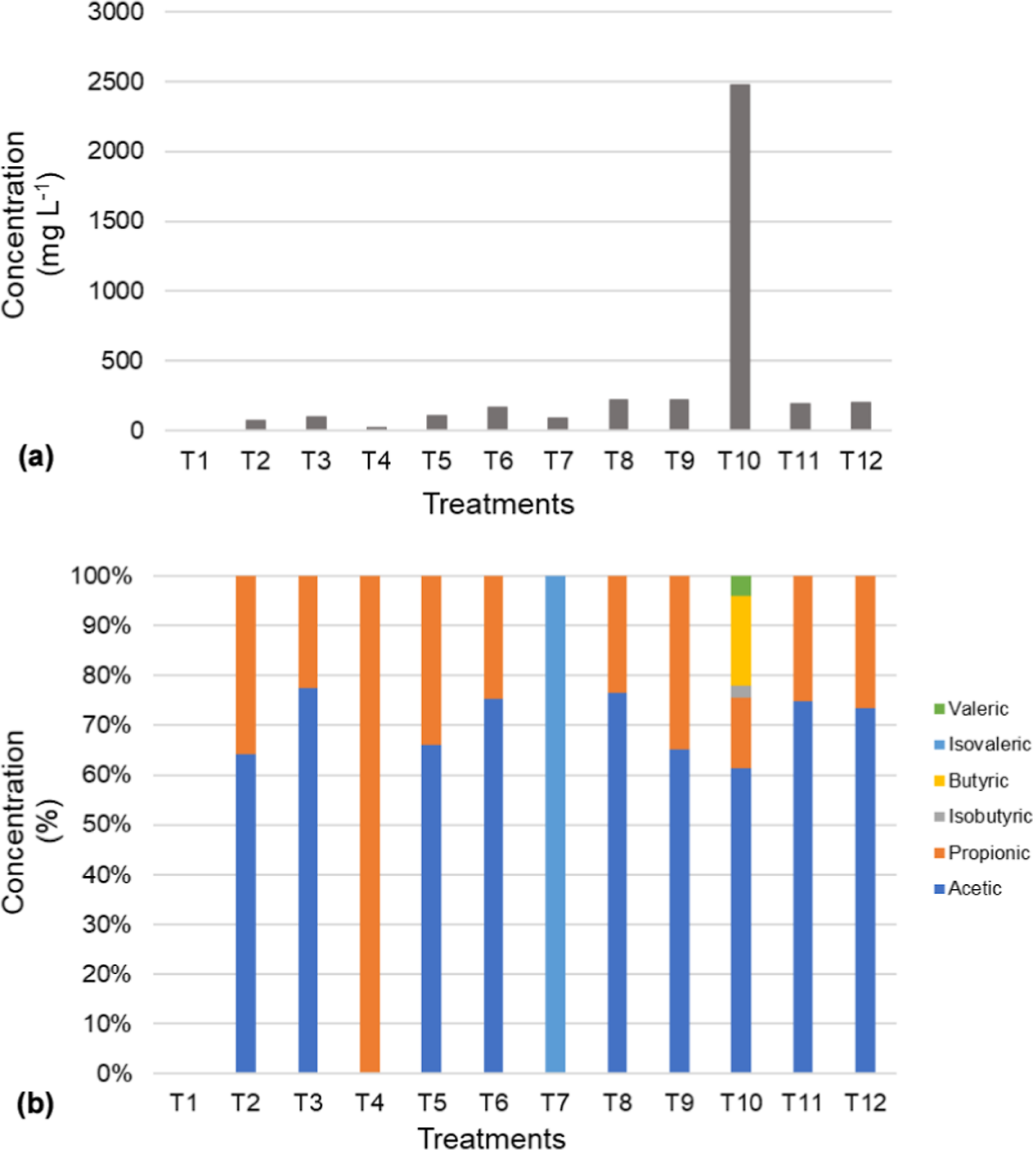
Absolute (a) and relative (b) concentrations of volatile fatty acids in the treatments.

On the other hand, treatment T10 presented a distinct scenario. With an S/I ratio of 2.0, TS of 17.2%, and C/N ratio of 37/1, the system reached the highest VFA accumulation recorded in the experiment, totaling 2.475 mg L^–1^ with 1.518 mg L^–1^ of acetic acid, 353 mg L^–1^ of propionic acid, 445 mg L^–1^ of butyric acid, and 101 mg L^–1^ of valeric acid being highlighted (Figure 5b). This VFA accumulation indicates a metabolic failure attributed to organic overload and lack of nutritional balance.^48^ This outcome, already noted in the article as a direct consequence of the adverse conditions in this treatment, compromised system stability and led to medium acidification, ultimately inhibiting methane production. Acetic acid was the predominant compound in nearly all treatments, as it is typically the final product of the acidogenic phase before conversion to methane.

### 3.4 Analysis of Microbiological Composition

Samples T1 and T4 were analyzed for their taxonomic composition by next-generation sequencing of 16S rRNA gene amplicons, which yielded 46,511 (T1) and 33,797 (T4) nonchimeric filtered reads. The bacterial phylum *Firmicutes* was the most representative in both samples (Figure 6), accounting for 86% in T1 and 82% in T4. Microorganisms from the *Firmicutes* phylum have the ability to release extracellular enzymes that promote the degradation and hydrolysis of residues with a high cellulose content. *Chloroflexi* was the second most predominant phylum in T1 (6%), and *Proteobacteria* was the second most predominant phylum in T4 (9%). The *Chloroflexi* phylum is capable of degrading a variety of complex macromolecules and recalcitrant substrates that slowly release organic matter such as lignocellulosic biomass. *Proteobacteria* are responsible for the degradation of cellulose and proteins as well as for the syntrophic degradation of organic acids. This group has been reported in acidic anaerobic digestion (AD) conditions, as observed in the accumulation of propionic acid (Figure 5b) in the T4 trial, which had a higher inoculum-to-substrate ratio and a higher C/N ratio. *Bacteroidota* was the third most representative phylum in both samples and contributed positively to the efficient degradation of cellulose. Regarding domain *Archaea*, phylum *Halobacterota* (0.4%) was identified in both samples (Figure 6). The proportion of archaea in the AD of lignocellulosic substrates is generally low (<1%) since bacterial hydrolysis is typically considered the rate-limiting step of the process.^49^

**Figure 6:**
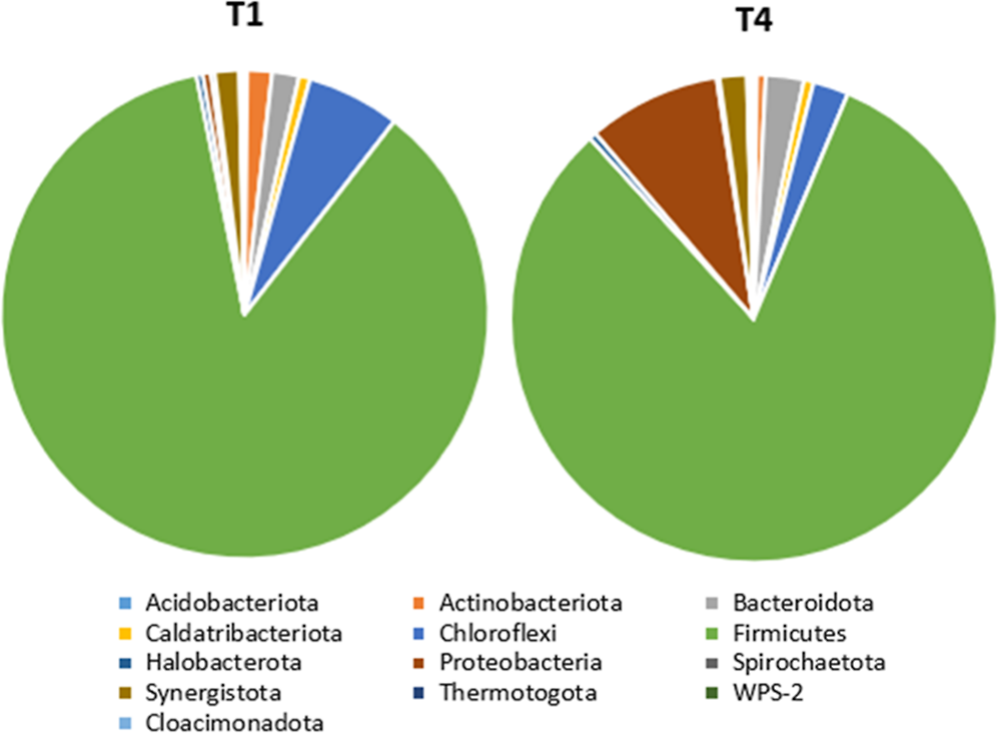
Taxonomic profile of the bacteria and archaea phyla present in T1 and T4.

Over 150 genera were identified in T1 and T4, but only 12 and 14 of these, respectively, were present in proportions greater than 1%. Taxonomic profile differences were observed between T1 and T4 (Figure 7), resulting solely from changes in the substrate-to-inoculum (S/I) ratio and, consequently, higher carbon and nitrogen concentrations. Among the identified genera, *Clostridium_sensu_stricto_1* was dominant in both samples (27% in T1; 20% in T4). This genus is ubiquitous in anaerobic digestion systems and can efficiently convert various carbon sources, such as lignocellulosic compounds, to acetic acid, butyric acid, H_2_, and CO_2_. The genus *Solibacillus* also showed high abundance in T1 (25%) and T4 (15%) and has been described as a lignocellulose degrader in AD processes using rice straw. The genera *Terrisporobacter* (3%) and *Rombousia* (2%) were prominent in T1 and are known to promote the production of volatile fatty acids (VFA). Meanwhile, *Sporosarcina* (6%), *Lysinibacillus*, and *MBA03* (3%) were more prevalent in T4 and are also involved in hydrolysis and fermentation processes related to VFA production. *Pseudomonas*, an electroactive bacterial species capable of oxidizing propionate and butyrate through extracellular electron transfer, was notable in T4 (6%), where an accumulation of propionic acid occurred.

**Figure 7:**
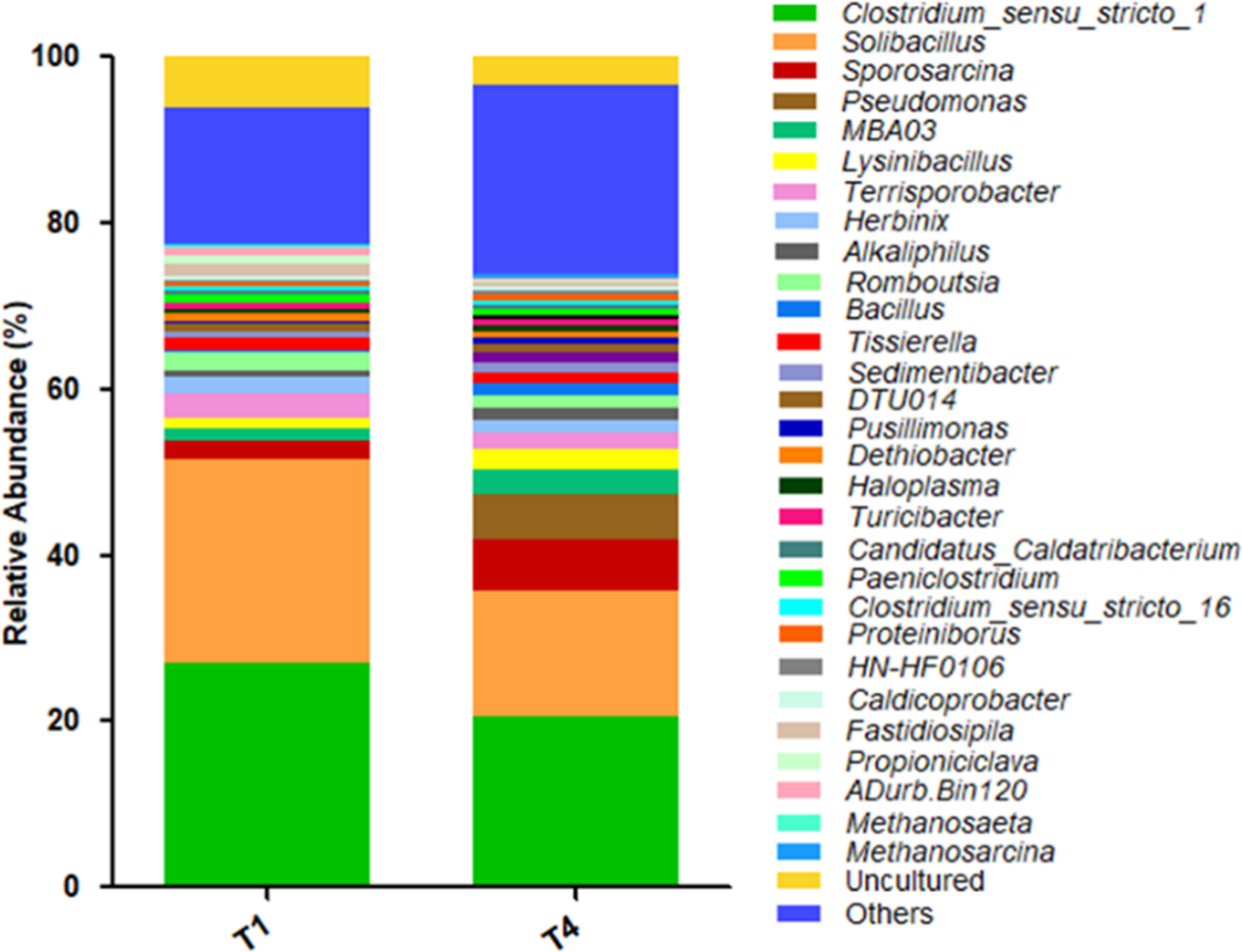
Dynamics of the relative abundance of bacterial and archaeal genera presence in T1 and T4.

The methanogenic archaea identified in T1 were predominantly *Methanosaeta* (0.3%), followed by *Methanosarcina* (0.10%) and *Methanospirillum* (0.02%). *Methanosaeta* (*Halobacterota*) is more sensitive to environmental factors such as free ammonia, pH, and temperature and uses only acetate to produce methane. This aligns with the total VFA consumption observed in this trial (Figure 5b) and the higher abundance of KO and genes involved in the acetoclastic metabolic pathway in T1 (Figure S1, Supporting Information). In contrast, AD in the T4 trial was dominated by *Methanosarcina* (0.32%), *Methanosaeta* (0.13%), *Methanospirillum* (0.01%), and *Methanobrevibacter* (0.08%). *Methanosarcina* is more resilient to environments disturbed by the VFA accumulation. It is a metabolic “generalist”, capable of using acetate, hydrogen, CO_2_, methanol, and methylamines as substrates, and shows greater resistance to adverse environmental conditions. In T4, there was a predominance of the hydrogenotrophic metabolic pathway (Figure S1). However, due to the low abundance of *Methanosarcina*, this may have led to H_2_ accumulation, creating an environment with excessive hydrogen partial pressure, which in turn severely inhibited propionic acid-degrading bacteria and led to its accumulation (Figure 5b). The methylotrophic metabolic pathway showed a low abundance of detected KOs.

## 4 Conclusions

Optimizing the substrate-to-inoculum ratio, total solids content, and carbon-to-nitrogen balance significantly improved the methane yield and process stability in SS-AD of sugar cane bagasse and bovine manure. Conditions of TS ≈12%, C/N ≈20–26, and S/I ≤1.5 optimized methane productivity while preventing the accumulation of inhibitory compounds. The optimized conditions (T4) resulted in 246 ± 6 NL_CH_4__ kg_VS_^–1^, which was equivalent to 233 ± 6 NL_CH_4__ kg_residue_^–1^ and 12.6 ± 0 L_CH_4__ L_reactor_^–1^ with 62% methane in the biogas composition. Microbial profiling demonstrated that the predominance of *Clostridium_sensu_stricto_1* and *Methanosarcina* supports both hydrolytic efficiency and methanogenic robustness under the optimized conditions. The results of this study reflect broader principles of SS-AD of lignocellulosic biomass. The integration of operational conditions with physicochemical and microbial analyses helps explain the mechanisms related to process stability and failure, supporting better design and scale-up of dry-digestion systems. Future studies should investigate the use of structural additives and microbial bioaugmentation to enhance mass transfer and scalability for industrial applications.

## Supplementary Material



## Notes

Experimental conditions of the experimental test and KO principles related to the metabolic pathways of methanogenesis to T1 and T4 (PDF)

## Abbreviations

AD: Anaerobic digestion
SCB: Sugar cane bagasse
BM: Bovine manure
GHG: Greenhouse gases
C/N: Carbon-to-nitrogen ratio
S/I: Substrate/inoculum ratio
TS: Total solids
VS: Volatile solids
TKN: Total Kjeldahl nitrogen
TAN: Total ammonia nitrogen
FA: Free ammonia
TOC: Total organic carbon
SS-AD: Solid-state anaerobic digestion
LD: Liquid digestate
SD: Solid digestate
TA: Total alkalinity
IA: Intermediate alkalinity
PA: Partial alkalinity
VFA: Volatile fatty acids
INO_mix_: Mixed inoculum
