# Supplementary material for: Methane Yield and Microbial Dynamics in Solid-State Anaerobic Codigestion of Sugar Cane Bagasse and Bovine Manure
Source: ACS Omega. 2026 Feb 19;11(8):13149–59. doi: 10.1021/acsomega.5c08172 (PMC12961475; doi:10.1021/acsomega.5c08172)

METHANE YIELD AND MICROBIAL DYNAMICS IN SOLID-STATE ANAEROBIC CO-DIGESTION OF SUGARCANE BAGASSE AND BOVINE MANURE

Larissa Maria Silveira Pereira[1], Carlos Vitor Ribeiro Pereira[1], Ilton José Baraldi[1], Igor Vinicius Machado Sophiatti[2], Flaviane Eva Magrini[2], Suelen Paesi[2], Prasad Kaparaju[3] e Thiago Edwiges[1*]

[1]Federal University of Technology – Paraná (UTFPR). Av., 4232 – Parque Independência, Medianeira/Brazil, 85884-000.

[2]University of Caxias do Sul (UCS), Molecular Diagnostic Laboratory, Biotechnology Institute, 95070-560, Caxias do Sul, RS, Brazil.

[3]Griffith University.170 Kessels Rd, Nathan QLD 4111, Australia

[*]Corresponding author: thiagoe@utfpr.edu.br

SUPPLEMENTARY MATERIAL

Table S1 - Experimental conditions of the experimental test.

Treatments	Substrate/inoculum	TS (%)	C/N	SCB:BM (w/w)	Organic matter concentration (g_{VS} L^{-1})
T1	0.5	10.0	21/1	100:0	67.0
T2	0.5	9.4	18/1	50:50	68.7
T3	0.5	8.9	16/1	0:100	70.5
T4	1.0	12.5	26/1	100:0	72.1
T5	1.0	11.1	21/1	50:50	75.5
T6	1.0	10.1	17/1	0:100	78.7
T7	1.5	14.9	32/1	100:0	75.9
T8	1.5	12.4	23/1	50:50	79.9
T9	1.5	10.9	18/1	0:100	84.6
T10	2.0	17.2	37/1	100:0	78.5
T11	2.0	13.4	25/1	50:50	83.7
T12	2.0	11.6	19/1	0:100	89.1

TS: total solids; C/N: Carbon/nitrogen ratio; SCB:BM: sugarcane bagasse: bovine manure ratio.

Figure S1: KO principals related to the metabolic pathways of methanogenesis to T1 and T4. Profile predicted with PICRUSt2 software based on KEGG reference database.

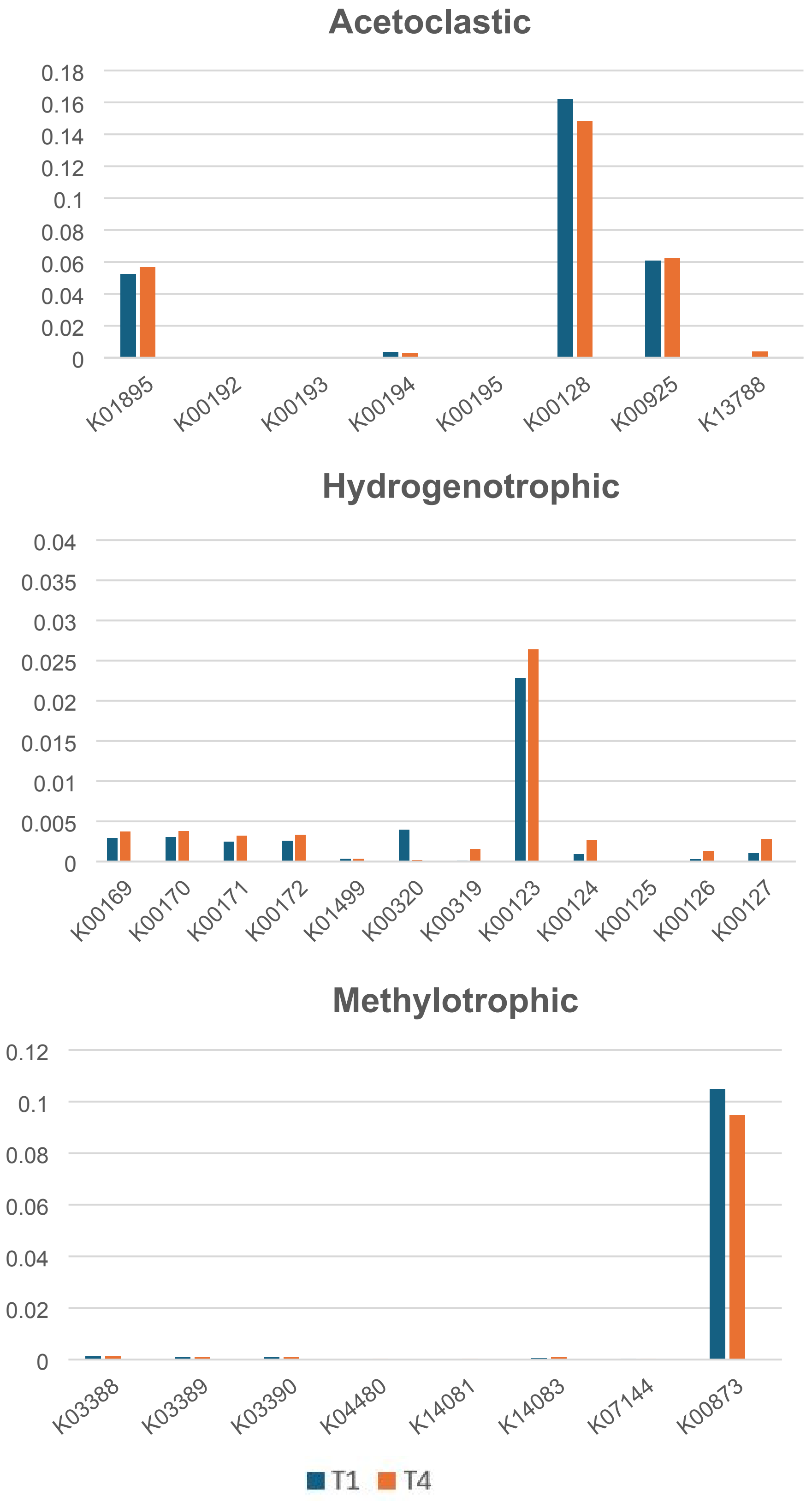

Supplement: Supplementary file 1 [file ao5c08172_si_001.pdf]
